# The microbiome and endometriosis

**DOI:** 10.1530/RAF-21-0113

**Published:** 2022-07-14

**Authors:** Carlos H Miyashira, Fernanda Reali Oliveira, Marina Paula Andres, Julian A Gingold, Mauricio Simões Abrão

**Affiliations:** 1 Centro de Ensaios Clinicos, Fundação Butantan, São Paulo, Sao Paulo, Brazil; 2 Departamento de Obstetricia e Ginecologia, Hospital das Clinicas HCFMUSP, Faculdade de Medicina, Universidade de Sao Paulo, Sao Paulo, Sao Paulo, Brazil; 3 Gynecologic Division, BP – A Beneficencia Portuguesa de Sao Paulo, Sao Paulo, Sao Paulo, Brazil; 4 OB/GYN and Women’s Health, Division of Reproductive Endocrinology and Infertility, Montefiore Medical Center, Bronx, New York, USA

**Keywords:** endometriosis, microbiome, systematic review, endometriosis stage.

## Abstract

The objective of this study was to systematically review the literature on the human microbiome in association with endometriosis. PubMed/Medline, Cochrane, and Embase databases were searched for literature published from 1986 to August 2021. All human studies that assessed the microbiome using 16S rRNA sequencing or shotgun sequencing in women with endometriosis were included. Two reviewers independently abstracted data from the selected articles into tables. To assess the quality of included studies, the National Institutes of Health Study Quality Assessment Tools were utilized. This review included 12 case–control studies. Included studies compared the microbiome from various anatomical sources (fecal, vaginal, cervical, peritoneal, endometrial, and intra-lesional) between patients with endometriosis and a heterogeneous set of control patients. Study quality ranged from poor to good, with 8 of 12 studies rated fair. Multiple studies reported a different distribution of bacteria among women with endometriosis across anatomical sites, but the results were highly heterogeneous. *Pseudomonas* was overrepresented in peritoneal fluid among women with endometriosis across multiple studies but was also observed to be increased in vaginal, endometrial, and intra-lesional samples. Among bacteria noted across different anatomical samples, *Gardnerella* was found to be increased in cervical but decreased in endometrial, fecal, and vaginal samples of patients with endometriosis, while *Atopium* was found to be decreased in vaginal and cervical samples from patients with endometriosis. *Sphingobium* was found to be increased in vagina, endometrium, and peritoneal fluid from patients with endometriosis. *Streptococcus* was found to be increased in peritoneal, endometrial, and cervical samples from women with endometriosis. Microbiomal comparisons stratified by endometriosis stage or site of endometriosis involvement were limited and highly heterogeneous.

**Lay summary:**

The microbiome, a group of bacteria found in a particular place in the body, has been shown to vary when patients have some diseases, such as cancer or inflammatory bowel disease. Less is known about the microbiome in patients with endometriosis. This review looked at existing studies comparing the bacteria found in patients with endometriosis and others without. Twelve studies were found that assessed the bacteria from swabs collected from different places, including the vagina, cervix, endometrium, peritoneum, feces, and endometriosis lesions themselves. Most of the studies found higher or lower levels of specific bacteria at each of these places, but the findings were often inconsistent. The findings were probably limited by the small numbers of patients involved and variations in the groups studied. More research is needed to find out which bacteria are over- and underrepresented in patients with endometriosis and where they are found.

## Introduction

Endometriosis is a multi-factorial disease defined by the presence of endometrial stroma or glands outside the uterine cavity. Patients with endometriosis, representing approximately 10–15% of reproductive-aged women, commonly experience dysmenorrhea, dyspareunia, and chronic pelvic pain, although there is a wide range in symptom prevalence as well as disease severity (Dunselman *et al.* 2014).

The most accepted theory on endometriosis pathogenesis is retrograde menstruation, in which reflux of menstrual blood through the fallopian tubes during menstrual cycles associated with an abnormal peritoneal environment permits the implantation and growth of ectopic endometrial tissue (Burney & Giudice 2019).

The advent of genomics technologies has greatly facilitated the characterization of the bacterial environment from clinical specimens with granular species-level detail. Previous studies have demonstrated that the microbiome may affect the development and progression of various diseases associated with an abnormal immune/inflammatory response, including inflammatory bowel diseases (Yang *et al.* 2021), autoimmune diseases (Tsai *et al.* 2021), and cancer (Lim *et al.* 2021, Pothuraju *et al.* 2021).

It is unknown whether an altered microbiome at any anatomical site can cause the development or progression of endometriosis. Similarly, it is not known whether endometriosis can directly induce an altered microbiome. Khan *et al*. (Khan *et al.* 2018) proposed a ‘bacterial contamination hypothesis’ for endometriosis, whereby bacterial endotoxins activate a peritoneal pro-inflammatory response, increase cell-to-cell adhesion, and facilitate the growth of ectopic endometrial implants.

Endometriosis most commonly occurs at sites such as the peritoneal cavity that are traditionally assumed to be sterile. However, microbiomal studies have also investigated swabs collected from sites known to have significant bacterial colonization, such as the vagina or rectum (Chen *et al.* 2017, Wang *et al.* 2021). Previous reviews (Leonardi *et al.* 2020, D’Alterio *et al.* 2021) have sought to investigate the association between endometriosis and the microbiome from different locations. For this systematic review, we considered microbiome analyses of swabs collected from all potential anatomical sites, regardless of whether the site was locally affected by endometriosis, and also sought to comprehensively collect data on endometriosis stage, menstrual phase, hormonal intake, and endometriosis symptoms.

## Objective

The primary objective was to systematically review the association between endometriosis and an altered microbiome across various anatomical sites. Secondary objectives were to evaluate the association between the endometriosis stage or pain symptoms and the microbiome.

## Methods

### Search strategy

Briefly, a literature search was performed on PubMed/Medline, Cochrane, and Embase databases from 1986 to August 2, 2021, using a combination of the following keywords: (microbiome OR microbial OR microbiota) AND (endometriosis OR endometrioma), and only articles published in English were considered. The full search strategy, including a dictionary of synonyms for the above keywords, is described in Supplementary Appendix 1 (see section on supplementary materials given at the end of this article).

### Selection criteria

All studies utilizing human subjects that assessed a bacterial microbiome in association with patients with endometriosis were included. Only case–control studies using semi-quantitative methodologies (such as 16s rRNA amplification or shotgun sequencing) capable of quantifying the relative bacterial prevalence between groups were included. Case reports, reviews, conference abstracts, animal studies, and unpublished studies were excluded from this review.

### Study selection

Two reviewers (FRO and CHM) independently screened the studies. Conflicts regarding study inclusion were resolved after a discussion between the two reviewers with a third author (MPA) and a senior author (MSA). Reviewers were not blinded to author names, institutional affiliations, or journal identities.

### Data abstraction

Two reviewers (FRO and CHM) independently abstracted data from the selected articles into tables. The following data were extracted for each study: author, year of publication, study design, comparison, sample size, endometriosis type (superficial, ovarian, and deep endometriosis), American Society of Reproductive Medicine (ASRM) stage (Revised American Society for Reproductive Medicine classification of endometriosis: 1996 1997), and results. When data were missing from the manuscript, efforts were made by two of the authors (CHM and FRO) to contact the corresponding authors to obtain complete data.

### Risk of bias

To assess the quality of included studies, publicly available study quality assessment tools provided by the National Institutes of Health (NIH) National Heart, Lung and Blood Institute were utilized, with specific forms for case–control and prospective non-randomized studies (https://www.nhlbi.nih.gov/health-topics/study-quality-assessment-tools). Conflicts regarding study quality were resolved with the senior authors (MPA and MSA). Studies that fulfilled 70% or more criteria were classified as good, 30–70% as fair, and less than 30% as poor quality.

### Statistical analysis

Studies were summarized and described qualitatively. Due to the heterogeneity of included studies, meta-analysis was not performed.

## Results

### Study selection

**Figure 1:**
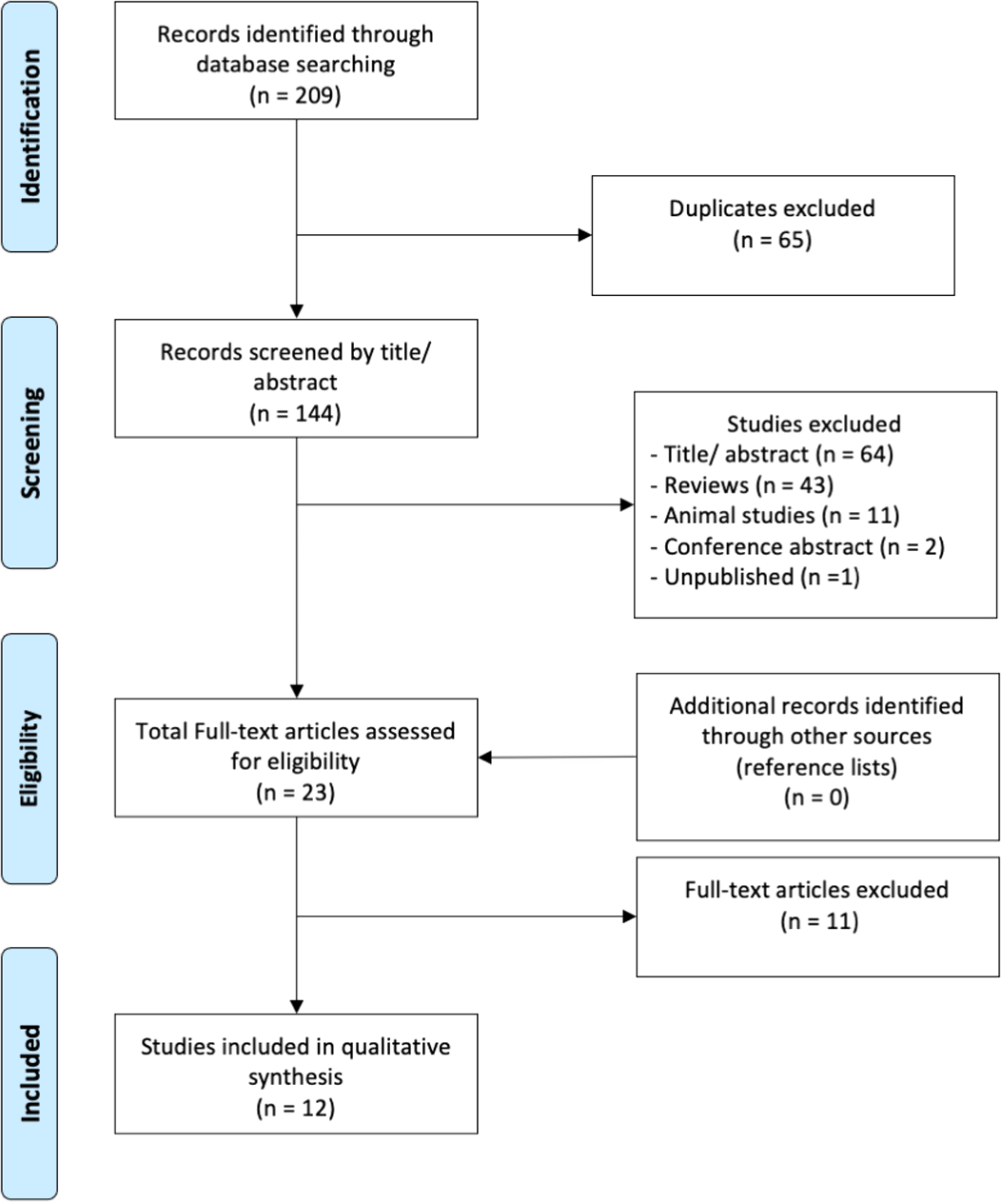
Flowchart of included studies.

Using the search strategy described above, the initial search identified 209 studies. After excluding 65 duplicates, 122 of the remaining 144 studies were excluded following title and abstract review. Full-text screening of 23 studies to evaluate for inclusion and exclusion criteria according to the study design, type of publication, methods, and results was performed by two authors (CHM and FOR), yielding 12 articles, all case–control studies (Khan *et al.* 2016, Xu *et al.* 2017, Wang *et al.* 2018, Akiyama *et al.* 2019, Ata *et al.* 2019, Chen *et al.* 2020, Hernandes *et al.* 2020, Perrotta *et al.* 2020, Wei *et al.* 2020, Chao *et al.* 2021, Lee *et al.* 2021, Svensson *et al.* 2021), meeting study inclusion criteria for data abstraction and qualitative analysis (Fig. 1). Key design characteristics of included studies are summarized in Table 1.

**Table 1:** Summary of included studies evaluating the microbiome and endometriosis.

Reference	Study design	*n*	Comparison	Age (years)	Sample	Methods
Akiyama *et al.* (2019)	Case–control	69	39 endometriosis	33.9 ± 5.7	Cervical	Ion Torrent Personal Genome Machine and qPCR
			30 controls: laparoscopy for myomas or benign ovarian tumors	32.5 ± 6.0		
Ata *et al.* (2019)	Case–control	28	14 endometriosis	28.6 ± 4.4	Stool, vaginal and cervical	Microbiome Shotgun sequencing
			14 controls: asymptomatic reproductive-aged women	27.8 ± 3.5		
Chao *et al.* (2021)	Case–control	128	37 endo/adeno with CPP (group A)	39.9 ± 6.2	Posterior vaginal fornix	Microbiome Shotgun sequencing
			25 controls with CPP (group B)	37.6 ± 5.5		
			66 controls without CPP (group C)	38.2 ± 7.8		
Chen *et al.* (2020)	Case–control	68	12 adenomyosis only, 13 endometriosis only, 7 both adenomyosis and endometriosis	36.1 ± 5.6	Cervical canal (67), posterior fornix (65), eutopic endometrium (2)	Microbiome Shotgun sequencing
			36 controls: infertility, myomas, ovarian borderline tumor, and teratoma			
Hernandes *et al.* (2020)	Case–control	21	10 endometriosis	18–50	Eutopic endometrium (18), endometriotic lesion (8), vaginal (21)	Microbiome Shotgun sequencing
			11 controls: laparoscopy for benign gynecologic diseases or elective tubal ligation			
Khan *et al.* (2016)	Case–control	64	32 endometriosis: with (16) or without (16) GnRHa	21–47	Eutopic endometrium, ovarian endometrioma fluid	Microbiome Shotgun sequencing
			32 controls: ovarian cyst or myoma, with (16) or without (16) GnRHa	21–52		
Lee *et al.* (2021)	Case–control	90	45 endometriosis	36.2 ± 1.3	Peritoneal fluid	Microbiome Shotgun sequencing
			45 controls: myomas (31) or benign ovarian cyst (14)	39.4 ± 1.1		
Perrotta *et al.* (2020)	Case–control	59	35 endometriosis	34.9 ± 6.8	Rectal and vaginal	Microbiome Shotgun sequencing
			24 controls: laparoscopy for benign gynecologic diseases	35.2 ± 6.9		
Svensson *et al.* (2021)	Case–control	264	66 endometriosis	38.0 ± 7.9	Stool	Microbiome Shotgun sequencing
			198 matched controls from the general population	37.7 ± 9.0		
Wang *et al.* (2018)	Case–control	85	55 endometriosis with infertility	37.2 ± 8.2	Peritoneal fluid	Microbiome Shotgun sequencing
			30 controls with infertility	37.7 ± 7.4		
Wei *et al.* (2020)	Case–control	50	36 endometriosis	23–44	Lower third of vagina, posterior vaginal fornix and cervical, eutopic endometrium, and peritoneal fluid	Ion Torrent Personal Genome Machine
			14 controls: laparoscopy for ovarian teratoma (7), serous cystadenoma (4), uterine myomas (3)			
Xu *et al.* (2017)	Case–control	10	5 endometriosis patients with chronic stress	31.8 ± 2.7	Stool	Microbiome Shotgun sequencing
			5 endometriosis without chronic stress	32 ± 4.1		

CPP, chronic pelvic pain; GnRHa, gonadotropin-releasing hormone agonist; LVFX, levofloxacin.

The endometriosis phenotypes of included patients were heterogeneous among studies and included all types of lesions (Khan *et al.* 2016, Xu *et al.* 2017, Wang *et al.* 2018, Chen *et al.* 2020, Perrotta *et al.* 2020, Wei *et al.* 2020), ASRM stages III–IV disease (Akiyama *et al.* 2019, Ata *et al.* 2019, Lee *et al.* 2021), deep endometriosis (Hernandes *et al.* 2020), and both ovarian and deep disease (Svensson *et al.* 2021).

### Study quality assessment and risk of bias

Eight studies were rated as fair (Khan *et al.* 2016, Xu *et al.* 2017, Wang *et al.* 2018, Akiyama *et al.* 2019, Ata *et al.* 2019, Chen *et al.* 2020, Wei *et al.* 2020, Lee *et al.* 2021), three as good (Hernandes *et al.* 2020, Perrotta *et al.* 2020, Svensson *et al.* 2021), and one as poor quality (Chao *et al.* 2021). Only one study (Perrotta *et al.* 2020) included a sample size justification and only one (Svensson *et al.* 2021) included concurrent controls. Researchers were not blinded in any of included studies (Tables 2 and 3). Owing to their case–control designs, no studies provided more than limited evidence (level 3b according to Oxford Center for Evidence-Based Medicine) for their findings.

**Table 2:** Quality assessment of case–control studies.

Question	Akiyama *et al.* (2019)	Ata *et al.* (2019)	Chao *et al.* (2021)	Chen *et al.* (2020)	Hernandes *et al.* (2020)	Khan *et al.* (2016)	Lee *et al.* (2021)	Perrotta *et al.* (2020)	Svensson *et al.* (2021)	Wang *et al.* (2018)	Wei *et al.* (2020)	Xu *et al.* (2017)
1. Was the research question or objective in this paper clearly stated and appropriate?	Y	Y	Y	Y	Y	Y	Y	Y	Y	Y	Y	Y
2. Was the study population clearly specified and defined?	Y	Y	Y	Y	Y	Y	Y	Y	Y	Y	Y	Y
3. Did the authors include a sample size justification?	N	N	N	N	N	N	N	Y	N	N	N	N
4. Were controls selected or recruited from the same or similar population that gave rise to the cases (including the same timeframe)?	Y	Y	N	Y	Y	Y	Y	Y	Y	Y	Y	Y
5. Were the definitions, inclusion and exclusion criteria, and algorithms or processes used to identify or select cases and controls valid, reliable, and implemented consistently across all study participants?	Y	Y	Y	Y	Y	Y	Y	Y	Y	Y	Y	Y
6. Were the cases clearly defined and differentiated from controls?	Y	Y	N	Y	Y	Y	Y	Y	Y	Y	Y	Y
7. If less than 100% of eligible cases and/or controls were selected for the study, were the cases and/or controls randomly selected from those eligible?	Y	Y	N	Y	Y	N/A	N/A	Y	N	N/A	Y	N/A
8. Was there use of concurrent controls?	N	N	N	Y	Y	Y	Y	Y	Y	Y	Y	N/A
9. Were the investigators able to confirm that the exposure/risk occurred prior to the development of the condition or event that defined a participant as a case?	N	N	N	Y	Y	Y	Y	Y	N/A	Y	Y	N/A
10. Were the measures of exposure/risk clearly defined, valid, reliable, and implemented consistently (including the same time period) across all study participants?	Y	Y	Y	Y	Y	Y	Y	Y	Y	Y	Y	Y
11. Were the assessors of exposure/risk blinded to the case or control status of participants?	N	N	N	N	N	N	N	N	N	N	N	N
12. Were key potential confounding variables measured and adjusted statistically in the analyses? If matching was used, did the investigators account for matching during study analysis?	N	N	N	N	N/A	N	N	Y	Y	Y	N	N
**Quality**	**Fair**	**Fair**	**Poor**	**Fair**	**Good**	**Fair**	**Fair**	**Good**	**Good**	**Fair**	**Fair**	**Fair**

Case–control studies were assessed using NIH study quality assessment tools. The replies represents if the study fulfilled each criteria (Y, yes; N, no: N/A, not applicable, not reported, or cannot determine). Overall study quality is summarized in the final row.

**Table 3:** Relative expression of bacterial loads in patients with endometriosis compared to patients without endometriosis. Summary of studies that evaluated the microbiome at different sites in patients with and without endometriosis. All studies compared relative frequencies of all bacteria reads performed by 16S RNA next generation sequencing.

Site	Decreased		Increased	
	Bacterial sp.	Reference	Bacterial sp.	Reference
Vagina	*Atopobium*	Ata *et al.* (2019)	*Aerococcus*	Wei *et al.* (2020)
	*Gardenerella*	Hernandes *et al.* (2020)	*Alloscardovia*	Chao *et al.* (2021)
	*Gemella*	Ata *et al.* (2019)	Atopobium*	Chen *et al.* (2020)
	*Lactobacillus*	Chao *et al.* (2021)	Campylobacter*	Chen *et al.* (2020)
	*Megasphaera*	Chao *et al.* (2021)	*Clostridium*	Chao *et al.* (2021)
	*Prevotella*	Hernandes *et al.* (2020)	*Escherichia*/ *Shigella*	Ata *et al.* (2019), Chen *et al.* (2020)
	*Shuttleworthia*	Chao *et al.* (2021)	Ezakiella*	Chen *et al.* (2020)
			Faecalibaterium*	Chen *et al.* (2020)
			*Gardnerella*	Ata *et al.* (2019)
			*Lactobacillus*	Chen *et al.* (2020)
			*Prevotella*	Wei *et al.* (2020)
			*Stenotrophomonas*	Chao *et al.* (2021)
			*Veillonella*	Chao *et al.* (2021)
Cervix	*Atopobium*	Ata *et al.* (2019)	*Comamonadaceae*	Wei *et al.* (2020)
	*Dialister*	Ata *et al.* (2019)	*Delftia*	Wei *et al.* (2020)
	*Megasphaera*	Ata *et al.* (2019)	*Enterobacteriaceae*	Akiyama *et al.* (2019)
	*Prevotella*	Ata *et al.* (2019)	*Escherichia*/ *Shigella*	Ata *et al.* (2019), Chen *et al.* (2020)
	*Snethia*	Ata *et al.* (2019)	*Pseudomonas*	Wei *et al.* (2020)
	*Snethia*	Ata *et al.* (2019)	*Sphingobium* spp	Wei *et al.* (2020)
			*Streptococcus*	Ata *et al.* (2019), Akiyama *et al.* (2019)
			*Ureaplasma*	Ata *et al.* (2019)
			*Vagococcus*	Wei *et al.* (2020)
Fecal	*Barnesella*	Ata *et al.* (2019)	*Lachnospira*	Svensson *et al.* (2021)
	*Gardnerella*	Ata *et al.* (2019)	*Oscillospira*	Svensson *et al.* (2021)
	*Snethia*	Ata *et al.* (2019)		
Endometrium	*Gardnerella*	Hernandes *et al.* (2020)	*Acinetobacter*	Wei *et al.* (2020)
	*Prevotella*	Hernandes *et al.* (2020)	*Delftia*	Wei *et al.* (2020)
			*Moraxellaceae*	Khan *et al.* (2016)
			*Pseudomonas*	Wei *et al.* (2020)
			*Sphingobium*	Wei *et al.* (2020)
			*Streptococcaceae*	Khan *et al.* (2016)
Lesion			*Alishewanella*	Hernandes *et al.* (2020)
			*Enterococcus*	Hernandes *et al.* (2020)
			*Pseudomonas*	Hernandes *et al.* (2020)
Peritoneal Fluid	*Actinomyces*	Lee *et al.* (2021)	*Acinetobacter guillouiae*	Wei *et al.* (2020), Lee *et al.* (2021)
	*Propionibacterium*	Lee *et al.* (2021)	*Clostridiales*	Wei *et al.* (2020)
	*Rothia*	Lee *et al.* (2021)	*Enhydrobacter*	Lee *et al.* (2021)
			*Erysipelothrix* sp.	Wei *et al.* (2020)
			*Pseudomonas viridiflava*	Wei *et al.* (2020), Lee *et al.* (2021)
			*Shewanella* sp.	Wei *et al.* (2020)
			*Sphingobium*	Wei *et al.* (2020)
			*Sphingomonas* sp.	Wei *et al.* (2020)
			*Streptococcus*	Lee *et al.* (2021)
			*Tissierellaceae*	Wei *et al.* (2020)

*Only on both endometriosis and adenomyosis group.

### Methods of evaluation of microbiome

Two next-generation sequencing (NGS) techniques were used to evaluate microbiomes: microbiome shotgun sequencing (Khan *et al.* 2016, Xu *et al.* 2017, Wang *et al.* 2018, Ata *et al.* 2019, Chen *et al.* 2020, Hernandes *et al.* 2020, Perrotta *et al.* 2020, Chao *et al.* 2021, Lee *et al.* 2021, Svensson *et al.* 2021) and Ion Torrent Personal Genome Machine (Akiyama *et al.* 2019, Wei *et al.* 2020). Akiyama *et al.* (2019), real‐time PCR was also used for quantification of *Enterobacteriaceae*, *Streptococcus*, *Pseudomonas*, and *Corynebacterium* genus.

Studies using NGS techniques analyzed different amplified regions of 16s-rRNA, including V1–V3 (Svensson *et al.* 2021), V3–V4 (Xu *et al.* 2017, Ata *et al.* 2019, Chen *et al.* 2020, Hernandes *et al.* 2020, Lee *et al.* 2021), V4 (Perrotta *et al.* 2020, Chao *et al.* 2021), V4–V5 (Wang *et al.* 2018, Wei *et al.* 2020), or V5–V6 (Khan *et al.* 2016, 2021, Xu *et al.* 2017, Wang *et al.* 2018, Akiyama *et al.* 2019, Ata *et al.* 2019, Chen *et al.* 2020, Hernandes *et al.* 2020, Perrotta *et al.* 2020, Wei *et al.* 2020, Chao *et al.* 2021, Lee *et al.* 2021, Svensson *et al.* 2021). One study (Khan *et al.* 2016) did not specify the rRNA amplification region.

### Control cohorts utilized for microbiome analysis

Eleven studies (Khan *et al.* 2016, Wang *et al.* 2018, Ata *et al.* 2019, Akiyama *et al.* 2019, Chen *et al.* 2020, Hernandes *et al.* 2020, Perrotta *et al.* 2020, Wei *et al.* 2020, Chao *et al.* 2021, Lee *et al.* 2021, Svensson *et al.* 2021) compared the microbiome between patients with and without endometriosis and one (Xu *et al.* 2017) compared endometriotic patients with and without chronic stress. In these studies, the control groups comprised patients who underwent surgery for other benign gynecological conditions (Khan *et al.* 2016, Akiyama *et al.* 2019, Chen *et al.* 2020, Hernandes *et al.* 2020, Lee *et al.* 2021, Perrotta *et al.* 2020, Wei *et al.* 2020), infertility (Wang *et al.* 2018), or chronic pelvic pain (CPP) (Chao *et al.* 2021) or asymptomatic patients who presented for routine gynecologic (Ata *et al.* 2019, Chao *et al.* 2021) or general visits (Svensson *et al.* 2021). The relative expression of bacteria across anatomical sites in patients with endometriosis compared to those without endometriosis is summarized in Table 3.

### Female reproductive tract microbiome and endometriosis

Seven studies evaluated the microbiome in vaginal and cervical samples (Akiyama *et al.* 2019, Ata *et al.* 2019, Chen *et al.* 2020, Hernandes *et al.* 2020, Perrotta *et al.* 2020, Wei *et al.* 2020, Chao *et al.* 2021) and three (Khan *et al.* 2016, Hernandes *et al.* 2020, Wei *et al.* 2020) in endometrial samples. Akiyama *et al.* (2019) performed a case–control study comparing 39 moderate-to-severe endometriosis patients against 30 patients with benign gynecological conditions undergoing surgery and found that the cervical microbiota was similar between the two groups. *Lactobacilli* species were predominant in both groups whereas *Enterobacteriaceae* and *Streptococcus* were more prevalent in women with endometriosis (*P* < 0.05).

Chen *et al.* (2020) compared the cervical and vaginal microbiome in 68 Chinese women stratified by the presence of endometriosis and adenomyosis and defined 4 groups: no endometriosis or adenomyosis, endometriosis only, adenomyosis only, and both adenomyosis and endometriosis (*n*  = 36, 13, 12 and 7, respectively). *Lactobacillus* was the most prevalent genus in the vagina in all groups, but the genus *Atopobium* was more commonly identified in women with both endometriosis and adenomyosis. *Campylobacter*, *Ezakiella*, and *Faecalibaterium* were also more abundant among patients with both endometriosis and adenomyosis.

Ata *et al.* (2019) studied the cervical and vaginal microbiome of 28 Caucasian women (14 with endometriosis ASRM stages III–IV and 14 asymptomatic patients without endometriosis who presented for a routine gynecological visit). They found that women with endometriosis were more likely to harbor *Alloprevotella* in the cervix, while *Atopobium* and *Sneathia* were only identified in the controls. *Gemella* and *Atopobium* were not detected in the vaginal microbiomes of endometriosis patients. When excluding *Lactobacillus* from the analysis, the relative abundance of *Gardnerella*, *Streptococcus*, *Escherichia*/*Shigella*, and *Ureaplasma* was found to be increased in endometriosis patients.

Hernandes *et al.* (2020) compared vaginal fluid and endometrial samples between 10 women with deep endometriosis and 11 without endometriosis undergoing benign gynecological surgery. While *Lactobacillus* predominated in the vaginal fluid of both endometriosis and control patients, *Gardnerella* and *Prevotella* were in lower relative abundance in samples of vaginal fluid and endometrium from endometriosis patients.

Perrotta *et al.* (2020) conducted an observational study comparing 35 Brazilian women with endometriosis stages I–IV against 24 without endometriosis undergoing surgery for benign gynecological diseases. The authors found no significant differences in the vaginal and rectal microbiome between endometriosis and control patients.

Wei *et al.* (2020) compared vaginal and cervical swabs from 16 Chinese women with stage I–II and 20 III–IV endometriosis against 14 women undergoing surgery for benign gynecological diseases. While the lower reproductive tract of both groups was dominated by *Lactobacillus, Aerococcus,* and *Prevotella* were enriched in endometriosis patients. Cervical swabs demonstrated enrichment of *Vagococcus*, *Arthrobacter*, *Pseudomonas*, *Sphingobium*, *Comamonadaceae,* and *Delftia* in women with endometriosis. Endometrial samples showed enrichment of *Sphingobium*, *Pseudomonas*, *Delftia*, and *Acinetobacter*.

Chao *et al.* (2021) compared 128 samples from the posterior vaginal fornix of Chinese women and divided them into 3 groups: 37 women with CPP plus endometriosis or adenomyosis, 25 women with CPP without endometriosis/adenomyosis, and 66 without CPP with endometriosis/adenomyosis who presented for a routine gynecologic visit. The group with endometriosis/adenomyosis and associated CPP was associated with a greater relative abundance of bacteria of the genera *Clostridium, Alloscardovia,* *Veillonella,* and *Stenotrophomonas* and a lower abundance of *Megasphaera*, *Lactobacillus,* and *Shuttleworthia* compared to those without endometriosis.

Khan *et al.* (2016) identified 32 women with endometriosis stages I–IV and 32 without endometriosis who underwent benign gynecological surgery and compared the presence of 5 bacterial families in endometrial samples: *Lactobacillacae*, *Streptococcaceae*, *Staphylococaceae*, *Enterobacteriaceae*, and *Moraxellaceae*. In women with endometriosis, there was an increase in *Streptococcaceae* and *Moraxellaceae*.

### Peritoneal fluid microbiome

Three studies (Wang *et al.* 2018, Wei *et al.* 2020, Lee *et al.* 2021) analyzed the relationship between endometriosis and the microbiome within the peritoneal fluid, one of which (Wei *et al.* 2020) also collected samples from other sites.

Lee *et al.* (2021), compared 45 women with stages III and IV endometriosis (mean age: 36.2 ± 1.3 years old) against 45 controls who underwent laparoscopy, 31 for myomas and 14 for benign ovarian cysts (mean age: 39.4 ± 1.1 years old). At a genus level, there was a significant increase in *Acinetobacter*, *Pseudomonas*, *Streptococcus*, and *Enhydrobacter* in the endometriosis group compared to the control group (*P*  < 0.05), as well as a significant reduction in the genera *Propionibacterium*, *Actinomyces*, and *Rothia* (*P* <  0.05).

Wang *et al.* (2018) compared 55 individuals with endometriosis and infertility (mean age: 37.2 ± 8.2 years old) against 30 controls with infertility without endometriosis (mean age: 37.7 ± 7.4 years old). The main bacteria detected in the peritoneal fluid were *Proteobacteria* and *Firmicutes*, followed by *Actinobacteria*, *Bacteroides*, *Fusobacterium*, and *Tenericutes*. There was no statistically significant difference between endometriosis and control groups (*P*  > 0.05).

Wei *et al.* (2020) compared peritoneal fluid samples of 50 Chinese women, 36 with pelvic endometriosis and 14 who underwent laparoscopy for ovarian teratoma, serous cystadenoma, or uterine fibroids. They found a significant increase in *Pseudomonas* and *Sphingobium* in the peritoneal fluid of women with endometriosis.

### Fecal microbiome

Two case–control studies compared the fecal microbiome of women with and without endometriosis (Ata *et al.* 2019, Svensson *et al.* 2021). Svensson *et al.* (2021) included 264 patients, comparing 66 women with endometriosis and with 198 matched controls from a cohort of descendants participating in the Malmö Diet and Cancer Cardiovascular Cohort (MDC-CC). The analysis showed only three bacteria with a significant difference with higher abundance between endometriosis and control groups: *Lachnospira*, *Oscillospira*, and a genus in the order *Bacterioidales* (*P* <  0.05).

Ata *et al.* (2019) compared 14 women with endometriosis against 14 asymptomatic reproductive-aged women who presented for a routine well-woman visit or preconception counseling. They found that the relative abundance of bacteria in the genera *Sneathia*, *Barnesella*, and *Gardnerella* from stool samples of the endometriosis group was significantly decreased (*P* <  0.001).

### Endometriosis stage or type and microbiome

Three studies (Khan *et al.* 2016, Perrotta *et al.* 2020, Svensson *et al.* 2021) compared the microbiome between patients across different endometriosis types or stages (Table 4). Perrotta *et al.* (2020) showed that the vaginal microbiome during the menstrual phase was significantly different between patients with ASRM stages III–IV compared to stages I–II (*P*  = 0.019), which was not significantly different from the vaginal microbiome of control patients. Patients with ASRM stage III–IV endometriosis had vaginal microbiomes enriched for *Anaerococcus* compared with lower-stage patients.

**Table 4:** Microbiomal studies comparing menstrual cycle phase, hormonal intake, or endometriosis type.

Reference	*n*	Hormonal treatment (*n*)	Menstrual phase (*n*, proliferative/secretory)	Endometriosis type (*n*)	Comparison of symptoms
Akiyama *et al.* (2019)	69	No	Control (17/22) Endometriosis (16/14) No difference between menstrual phase	ASRM stages III–IV	Not reported
Ata *et al.* (2019)	28	No	Control (7/7) Endometriosis (7/7)	ASRM stages III–IV	Not reported
Chao *et al.* (2021)	128	Combined oral contraceptives (75) and IUD (11). No comparison between groups	Endometriosis/adenomyosis with CPP (12/15) Controls with CPP (5/16) Controls without CPP (22/35)	Not reported	↓Lactobacillus jensenii, ↓ Shuttleworthia, ↑ Clostridium butyricum, ↑ Alloscardovia in endometriosis patients with chronic pelvic pain
Chen *et al.* (2020)^*^	68	No	Not reported	Ovarian endometriosis, deep, and peritoneal	Not reported
Hernandes *et al.* (2020)	21	Yes^**^	Not reported	Deep endometriosis	Not reported
Khan *et al.* (2016)	64	GnRHa (16) ↓ Lactobacillacae, ↑*Streptococcaceae*, ↑*Staphylococaceae*, ↑*Enterobacteriaceae* in GnRHa-treated women with endometriosis vs untreated women. ↑*Staphylococaceae* in GnRH-treated compared with untreated control women	Control (4/10) Endometriosis (2/9)	ASRM stage I (11), II (2), III (7), and IV (12)	Not reported
Lee *et al.* (2021)	90	No	Not reported	ASRM stages III (34) and IV (11)	Not reported
Perrotta *et al.* (2020)	59	No	Menstrual and proliferative ↑ *Lactobacillus* in proliferative phase compared to secretory and menstrual	Bowel (13), retrocervical (14), bladder (4), ovarian (2), superficial (1), and abdominal wall (1). ASRM stages I (9), II (12), III (4), and IV (10).	Not reported
Svensson *et al.* (2021)	264	Yes (41) ↑ *Blautia,* ↑ *Ruminococcus,* ↑*Butyricimonas* among those taking hormones	Not reported	Ovarian (27), Gastrointestinal (18)	No significant association with the intensity of pain symptoms or digestive complaints
Wang *et al.* (2018)	85	No	Not reported	ASRM stages I–II (28) and stages III–IV (27)	Not reported
Wei *et al.* (2020)	50	No	Proliferative (50)	ASRM stage I–II (16) and stages III–IV (20)	Not reported
Xu *et al.* (2017)	10	Not reported	Not reported	ASRM stages I–II (2) and stages III–IV (8)	↓ Paraprevotella, ↓Odoribacter, ↓Veillonella ↓Ruminococcus, and ↑ *Prevotella* in chronically stressed endometriosis patients

^*^This study included four groups: no endometriosis or adenomyosis (*n* = 36), endometriosis only (*n* = 13), adenomyosis only (*n* = 12), and both adenomyosis and endometriosis (*n*  = 7). ^**^Number of patients taking hormones not reported.

ASRM, American Association for Reproductive Medicine Classification; CPP, chronic pelvic pain.

Two studies (Khan *et al.* 2016, Svensson *et al.* 2021) compared the fecal or ovarian cyst microbiome among different types of endometriosis without using the ASRM staging system. Svensson *et al.* (2021) found no significant difference in the stool microbiome between ovarian and deep endometriosis. Khan *et al.* (2016) found a significantly higher percentage of *Streptococcaceae* and *Staphylococaceae* and a significant reduction in *Lactobacillacae* in the ovarian endometrioma cystic fluid in comparison with non-endometriotic cysts.

### Microbiome and menstrual cycle phase

While six microbiomal studies among endometriosis patients (Khan *et al.* 2016, Akiyama *et al.* 2019, Ata *et al.* 2019, Wei *et al.* 2020, Perrotta *et al.* 2020, Chao *et al.* 2021) reported on the menstrual cycle phase, only two compared the microbiome during different menstrual phases (Table 4). Akiyama *et al.* (2019) found no significant differences in the cervical microbiome across different menstrual phases of either endometriosis or control patients. Perrotta *et al.* (2020) observed an increase in vaginal *Lactobacillus* species in the proliferative phase compared to the secretory and menstrual phases. The authors (Perrotta *et al.* 2020) also observed an increase in anaerobic bacteria in the endometrium or peritoneal fluid during the proliferative and secretory phases compared to the menstrual phase.

### Hormonal intake and microbiomal variation among endometriosis patients

Four studies (Khan *et al.* 2016, Hernandes *et al.* 2020, Chao *et al.* 2021, Svensson *et al.* 2021) included women possibly taking hormonal agents, while seven (Wang *et al.* 2018, Akiyama *et al.* 2019, Ata *et al.* 2019, Chen *et al.* 2020, Perrotta *et al.* 2020, Wei *et al.* 2020, Lee *et al.* 2021) were restricted to patients without current hormonal intake (Table 4). Khan *et al.* 2016 evaluated the effect of the use of a gonadotropin-releasing hormone agonist (GnRHa) on women with endometriosis and showed that *Lactobacillacae* was significantly decreased (*P*  < 0.01), while *Streptococcaceae*, *Staphylococaceae,* and *Enterobacteriaceae* were significantly increased (*P*  < 0.05 for each) in vaginal swabs from GnRHa-treated women with endometriosis compared with GnRHa-untreated women. In contrast, vaginal samples from GnRHa-treated control women showed significantly higher colonization with *Staphylococaceae* (*P*  < 0.05) and insignificant colonization with *Enterobacteriaceae* (*P*  = 0.071) compared with samples from GnRHa-untreated control women. Svensson *et al.* (2021) examined the fecal microbiome among women with endometriosis and identified a higher abundance of *Blautia*, *Ruminococcus*, and *Butyricimonas* among those taking hormonal medications, including estrogen, combined oral contraceptives, progestin, or gonadotropin-releasing hormone analogs. The remaining studies did not report on changes in the microbiome in association with hormonal intake.

### Endometriosis symptoms and microbiome

Three studies (Xu *et al.* 2017, Chao *et al.* 2021, Svensson *et al.* 2021) compared the association between endometriosis symptoms and the microbiome. Svensson *et al.* (2021) compared 66 patients with endometriosis and 198 asymptomatic women without endometriosis from the MDC-CC cohort described above. In a subanalysis of the 66 endometriosis patients, they reported no significant association of their stool microbiome with the intensity of pain symptoms or digestive complaints, including abdominal pain, constipation, diarrhea, bloating, and vomiting.

Xu *et al.* (2017) studied the fecal microbiome of ten subjects with endometriosis, five reporting chronic stress and five not reporting chronic stress. They found significantly decreased levels of *Paraprevotella*, *Odoribacter*, *Veillonella* and *Ruminococcus* in chronically stressed endometriosis patients, while *Prevotella* was significantly increased among the chronically stressed endometriosis patients.

Chao *et al.* (2021) compared the fecal microbiome of 37 patients with endometriosis or adenomyosis plus (CPP, 25 patients without endometriosis but reporting CPP, and 66 without endometriosis or CPP. Patients with endometriosis and CPP were found to have the lowest relative abundance of *Lactobacillus jensenii* and the highest abundance of *Clostridium butyricum* compared to the other two groups. Endometriosis patients with CPP also had significantly lower *Lactobacillus* and *Shuttleworthia* and significantly higher *Clostridiales* and *Alloscardovia* abundance compared with no endometriosis patients without CPP, but no difference compared to patients with CPP and without endometriosis.

## Discussion

This review identified multiple microbiome studies on patients with endometriosis. This systematic review highlighted many of the limitations of such studies, including heterogeneous methods for identifying and typing bacteria, various anatomical sources for microbiomal sample collection (fecal, vaginal, cervical, peritoneal, endometrial, and intra-lesional), significant heterogeneity among patients both with endometriosis and the so-called controls (including heterogeneity in menstrual cycle timing, use of hormonal medications, symptomatology, disease severity, and the presence of comorbid conditions such as adenomyosis), and inherent publication bias. Such limitations collectively have precluded completing a meta-analysis of the underlying studies.

Nonetheless, several trends appear to stand out from these imperfect, heterogeneous studies. Several studies suggest that peritoneal fluid appears to contain a different distribution of bacteria among women with endometriosis, though only *Pseudomonas* (Wei *et al.* 2020, Lee *et al.* 2021) was found to be overrepresented among patients with endometriosis in multiple studies. Fecal microbiome studies (Xu *et al.* 2017, Ata *et al.* 2019, Svensson *et al.* 2021) appear to be conflicting in the reported prevalence of various bacteria. The one study that reported an association between chronic stress in endometriosis and an altered fecal microbiome (Xu *et al.* 2017) is yet to be validated.

While the association between the fecal microbiome and endometriosis remains inconclusive, the topic remains biologically plausible. The gut microbiome interacts with immune and metabolic systems and is associated with various disease states, including inflammatory bowel syndrome, arthritis, psoriasis, and cancer (Smet *et al.* 2021, Wertman *et al.* 2021). The dysbiosis of the gastrointestinal tract can lead to higher gut permeability, a higher concentration of macrophages in peritoneal fluid, secretion of interleukin IL-1 and IL-10, and modulation of local immune response to the clearance of menstrual debris and thus potentiate endometriosis development (D’Alterio *et al.* 2021). Also, it has been suggested that dysbiosis of the gut microbiome may alter the so-called estrobolome and lead to enhanced estrogen deconjugation and increased free circulating levels, potentially contributing to endometriosis progression (García-Peñarrubia *et al.* 2020).

The inferior female reproductive tract is a major source of human microbiota, urogenital microbiota being responsible for 9% of all bacterial species in the human body (Cani 2018). Cervicovaginal lactobacilli deficiency is correlated with higher genital pro-inflammatory cytokines and activation of antigen-presenting cells through lipopolysaccharide (LPS) pathways (Cani 2018). Also, studies have shown that the fecal and vaginal microbiota are correlated and that the use of probiotics can impact both the fecal and vaginal environments, suppressing pro-inflammatory cytokine production (Melis *et al.* 2018).

While the diversity of the vaginal and fecal microbiome is well-recognized, the presence of meaningful bacterial colonization at other sites such as the endometrium or within endometriosis biopsies remains controversial. Identification of bacteria at supposedly sterile sites may suggest contamination or another infectious process rather than evidence of endometriosis (Chen *et al.* 2017). The upper genital tract may become colonized via the bloodstream, mesenteric lymph nodes, or through the retrograde progression of cervical and vaginal bacteria, though its role in modulating uterine health in unclear (Baker *et al.* 2018, Wang *et al.* 2021).

Previous studies suggested that the microbiome in the vaginal tract may be influenced by hormonal treatments and the menstrual cycle phase. Despite this, only two studies (Akiyama *et al.* 2019, Perrotta *et al.* 2020) attempted to address confounding from the menstrual phase. The lack of such standardization and correction for clear confounding variables is a significant limitation that should be addressed in future studies. Similarly, most studies did not attempt to control for the endometriosis stage, thus limiting the generalizability of observed results. For example, patients with endometriosis infiltrating the bowel have a much more plausible and direct connection to developing an altered fecal microbiome than patients with endometriosis without bowel involvement. Future prospective studies with larger samples and stricter methodology combined with patient standardization are needed to clarify the role of the microbiome in endometriosis pathogenesis and clinical features and allow for a precise measurement of the effect of any interventions.

## Conclusion

Clear differences have been reported from studies of the fecal, vaginal, cervical, endometrial, and peritoneal microbiomes of women with and without endometriosis. An association of the microbiome with the hormonal intake, menstrual cycle phase, and pain symptoms in patients with endometriosis was reported by a few studies. However, studies are limited due to a lack of standardization and small samples, and the cause–effect relationship between the microbiome and endometriosis is yet to be established.

## Supplementary Material

Appendix 1 – Search strategy utilized for this systematic review.

## Declaration of interest

The authors declare that there is no conflict of interest that could be perceived as prejudicing the impartiality of the research reported.

## Funding

This work did not receive any specific grant from any funding agency in the public, commercial, or not-for-profit sector.

## Author contribution statement

Carlos H Miyashira: study design, data collection, data analysis and interpretation, manuscript preparation. Fernanda Reali Oliveira: study design, data collection, data analysis and interpretation, manuscript preparation. Marina Paula Andres: study design, data analysis and interpretation, manuscript preparation. Julian A Gingold: manuscript preparation. Mauricio Simões Abrão: study design, manuscript preparation.
