# Supplementary material for: The microbiome and endometriosis
Source: Reprod Fertil. 2022 Jul 14;3(3):R163–75. doi: 10.1530/RAF-21-0113 (PMC9422234; doi:10.1530/RAF-21-0113)
Supplement: Appendix 1 – Search strategy utilized for this systematic review. [file supplementary_table_1.pdf]

Appendix 1 – Search strategy utilized for this systematic review.

Pubmed/Medline

("endometriosis"[MeSH Terms] OR "endometriosis"[All Fields] OR "endometrioses"[All Fields] OR ("endometriosis"[MeSH Terms] OR "endometriosis"[All Fields] OR "endometrioma"[All Fields] OR "endometriomas"[All Fields])) AND ("microbiome s"[All Fields] OR "microbiomic"[All Fields] OR "microbiomics"[All Fields] OR "microbiota"[MeSH Terms] OR "microbiota"[All Fields] OR "microbiome"[All Fields] OR "microbiomes"[All Fields] OR ("microbial"[All Fields] OR "microbially"[All Fields] OR "microbials"[All Fields]) OR ("microbiota"[MeSH Terms] OR "microbiota"[All Fields] OR "microbiotas"[All Fields] OR "microbiota s"[All Fields] OR "microbiotae"[All Fields]))

Cochrane library: endometriosis AND microbiome

Embase: endometriosis [all fields] AND microbiome [all fields]
